# Real-World Data of Patients with *BRAF* V600E-Mutated Metastatic Colorectal Cancer Treated with Trifluridine/Tipiracil

**DOI:** 10.3390/cancers16244140

**Published:** 2024-12-12

**Authors:** Javier Ros, Jose Maria Ucha, Eduardo Garcia-Galea, Pablo Gomez, Giulia Martini, Francesca Balconi, Raquel Comas, Vicente Alonso, Marta Rodriguez, Iosune Baraibar, Francesc Salva, Nadia Saoudi, Adriana Alcaraz, Ariadna Garcia, Josep Tabernero, Elena Elez

**Affiliations:** 1 Medical Oncology, Vall d’Hebron University Hospital, 08035 Barcelona, Spain; 2 Vall d’Hebron Institute of Oncology, 08035 Barcelona, Spain; 3 Medical Oncology, Miguel Servet Hospital, 50009 Zaragoza, Spain; 4 Department of Precision Medicine, University of Campania Luigi Vanvitelli, 80138 Naples, Italy; 5 Medical Oncology, University Hospital, University of Cagliari, 09124 Cagliari, Italy

**Keywords:** *BRAF* mutation, colorectal cancer, trifluridine/tipiracil

## Abstract

Background: For patients with refractory metastatic colorectal cancer (mCRC), trifluridine/tipiracil (FTD–TPI) has been associated with a significant improvement in overall survival (OS). However, data are lacking regarding the activity of FTD–TPI in patients with *BRAF*-mutated mCRC. Methods: This retrospective, multicenter, international cohort included patients with *BRAF*-mutated mCRC treated with FTD–TPI in a real-life setting in Spain and Italy. Survival analysis was performed using Kaplan–Meier methods and Cox proportional hazard models and according to established prognostic groups: good prognosis characteristics (GPC; < 3 metastatic sites and time from metastases to FTD–TPI ≥ 18 months) and poor prognosis characteristics (PPC; ≥ 3 metastatic sites or time from metastases to FTD–TPI < 18 months). Results: In the 26 patients included, the median age was 61 years, 13 (50%) were female, and 20 (77%) had an Eastern Cooperative Oncology Group (ECOG) performance status of 1. Fourteen (56%) patients had right-sided tumors, six (23%) had microsatellite instability tumors, and thirteen (50%) had liver metastases. Median progression-free survival was 2.3 months (95% CI 2.0–3.2), and median OS (mOS) was 6.6 months (95% CI 4.4–12.0). mOS was 7.6 vs. 4.2 months (HR 1.64, 95% CI 0.65–4.10, *p* = 0.3) for GPC and PPC patients, respectively. Exploratory analyses identified ECOG as the only feature associated with survival. The most frequent grade 3–4 adverse events were neutropenia (8%), anemia (8%), and asthenia (4%). Conclusions: Patients with *BRAF* mutant mCRC achieved modest benefits with FTD–TPI; however, patients with GPC and ECOG 0 achieved longer OS compared with those with PPC or ECOG 1–2, thus warranting further exploration in prospective cohorts.

## Simple Summary

Trifluridine/tipiracil (FTD–TPI) is a therapy for metastatic colorectal cancer after progression on standard treatment. Some patients with colorectal cancer harbor a *BRAF* gene mutation and may not respond as well to treatment compared to patients without this mutation. We evaluated patients with a *BRAF* mutated colorectal cancer treated with FTD–TPI as part of routine hospital care. Survival for these patients was 6.6 months, with disease progression after 2.3 months. Patients’ functional status (according to a standardized scale “ECOG”) stands as the most relevant factor guiding survival outcome; patients with a better status (ECOG 0) survived for longer than patients with poorer status (ECOG 2; 12 months vs. less than 2 months, respectively). Side effects with FTD-TPI were equivalent to those reported for the overall colorectal cancer population. This study suggests that FTD–TPI remains a therapeutic option for selected patients with *BRAF* mCRC.

## 1. Introduction

According to GLOBOCAN, colorectal cancer (CRC) is the third most common cancer worldwide, and up to 50% of patients with CRC will develop metastatic disease [1,2,3]. Mutations in *BRAF* occur in approximately 12% of metastatic CRC (mCRC) cases, with the *BRAF* V600E mutation being the most common (accounting for up to 95% of all *BRAF* mutations) [4]. The *BRAF* V600E mutation is associated with aggressive disease, poor response to standard chemotherapy, and reduced overall survival (OS), even when treated with targeted agents [5,6,7]. However, due to its low prevalence, specific data on clinical activity and safety are rarely reported for the *BRAF* subgroup in clinical trials. It remains important to characterize the safety and efficacy of approved therapies in *BRAF*-mutant mCRC.

In terms of personalized treatment, patients with *BRAF* V600E mCRC should not be treated with anti-EGFR agents either as monotherapy or in combination with chemotherapy due to the lack of additional benefit [7]. Conversely, subgroup analyses from several clinical trials combining anti-VEGF agents with chemotherapy have confirmed the benefit of adding bevacizumab, aflibercept, or ramucirumab [8,9,10,11]. In the refractory setting, the phase III BEACON trial demonstrated the benefit of the BRAF inhibitor encorafenib plus cetuximab with or without the MEK inhibitor binimetinib over irinotecan-cetuximab-based chemotherapy [12], and this treatment is now recommended as the standard of care for patients with refractory *BRAF* V600E mCRC [13]. However, and despite the development of several prognostic and predictive biomarkers [14,15,16], almost all patients eventually progress after BRAF inhibitor-based therapy [12,17]. Furthermore, due to the aggressiveness of *BRAF* V600E mCRC, only 30–50% of patients will be able to receive further treatment [18]. Therapeutic options after encorafenib-cetuximab treatment include regorafenib, trifluridine/tipiracil (FTD-TPI) with or without bevacizumab, and fruquintinib [19,20,21,22]. Nonetheless, no studies using these agents have provided data regarding clinical activity and safety for the *BRAF*-mutated population.

FTD-TPI is an orally active nucleoside antitumor agent that has been approved for refractory mCRC [21]. Notably, using the approach that better patient selection might lead to improved outcomes, an analysis from the RECOURSE trial (NCT01607957; phase III randomized trial comparing FTD-TPI versus placebo in patients with refractory mCRC) established two prognostic groups based on tumor burden and disease indolence. Patients with favorable prognostic characteristics (<3 metastatic sites and time from metastases to FTD–TPI ≥ 18 months) had better OS compared to those with poor prognostic characteristics (9.3 vs. 5.3 months) [23]. However, the efficacy of FTD-TPI in the *BRAF*-mutant population has not yet been reported [20,21,24]. Finally, the advent of the precision medicine paradigm in oncology has heightened interest in the integration of real-world data into cancer clinical research. As sources of real-world evidence, real-world data can address uncertainties surrounding the adoption of novel anticancer therapies into clinical practice after evaluation in randomized controlled trials. Currently, most real-world evidence-generating studies on antitumor interventions rely on observational real-world data, often foregoing randomization despite its methodological advantages [25]. We performed a retrospective study designed to analyze efficacy and safety data from patients with *BRAF* V600E mCRC treated with FTD-TPI in the real-world setting, including patients from two hospitals in Italy and two hospitals in Spain.

## 2. Materials and Methods

A retrospective, descriptive analysis was performed including patients with histologically confirmed *BRAF* V600E mutant mCRC aged 18 years or older who were treated at four hospitals in Spain and Italy with FTD-TPI as part of their routine care between 2016 and 2023. This retrospective study was approved by the institutional review board or independent ethics committee at each center and was conducted in accordance with the requirements of the regulatory authorities of each country (approval number EOM(AG)055/2023(6200)). Data obtained from medical records were analyzed based on tumor and patient characteristics, including sex, age, Eastern Cooperative Oncology Group (ECOG) status, tumor sidedness, mismatch repair (MMR) status, number of previous treatment lines, number of metastatic sites, presence of liver metastases, levels of carcinoembryonic antigen (CEA) and CA 19–9, time from diagnosis of metastatic disease to the start of FTD-TPI treatment, number of cycles administered, and dose modifications.

OS and progression-free survival (PFS) were evaluated in the total population and according to ECOG status and prognostic characteristics based on previously reported prognostic subgroups from the RECOURSE trial [23]: good prognostic characteristics (GPC) included patients with fewer than 3 metastatic sites and time from metastasis to the start of FTD-TPI ≥ 18 months, and poor prognostic characteristics (PPC) included patients with ≥3 metastatic sites or time from metastases to FTD-TPI < 18 months. The ESMO Guidance for reporting Oncology-real-World Evidence (ESMO-GROW) checklist is available in the Supplementary section.

Demographic and baseline characteristics of the patients were summarized by treatment group using descriptive statistics (n, mean, median, range) and/or frequency distributions, as appropriate. Baseline differences among prognostic groups were assessed using the most appropriate non-parametric test for each variable (Wilcoxon rank sum test, Pearson’s Chi-Squared test, or Fisher’s exact test). The Kaplan–Meier method and Cox proportional hazard regression models were used to determine OS and PFS in the overall population and for the ECOG subgroup and prognostic group to identify independent prognostic factors in univariate analyses. Potential prognostic biomarkers, including sex, age at diagnosis, tumor sidedness, previous lines of therapy, microsatellite instability (MSI) status, liver, peritoneal and lung metastases, ECOG performance status (PS), CEA, and CA19.9, were evaluated in a univariate analysis. All statistical analyses were carried out using R statistical software (Version 2023.09.0+463). Hazard ratios (HRs) with 95% confidence intervals (CIs) were reported. A *p*-value < 0.05 was considered statistically significant.

## 3. Results

Overall, 26 consecutive patients were included in the study. Baseline characteristics of the population are summarized in Table 1. Half of the patients were female, and the median age was 61 years. Most patients had ECOG PS 1 (77%). Right-sided tumors were present in 56% of the population, and 77% of all tumors were microsatellite-stable (MSS). Most patients (54%) had received three or more previous lines of treatment before starting FTD-TPI, and 35% of patients had three or more metastatic sites. Half of the patients had liver metastases. The median time from metastatic disease diagnosis to the start of FTD-TPI was 29 months, and 31% of patients received three or more cycles. Regarding dose modifications, 35% of patients required dose adjustments due to toxicity.

When analyzing the population by prognostic subgroups, 14 patients (54%) were allocated to the GPC subgroup, and 12 patients (46%) were included in the PPC subgroup. Among patients in the GPC subgroup, 69% had right-sided tumors, 29% had microsatellite instability (MSI), and 79% had received at least three previous lines of therapy. Regarding FTD-TPI treatment, 43% of GPC patients completed at least three cycles, and only 21% required dose modifications due to toxicity. Conversely, 58% of tumors in the PPC subgroup were left-sided, and 83% were MSS. Among PPC patients, only 25% had previously received three or more lines, while 75% had three or more metastatic sites. The median time from metastatic diagnosis to FTD-TPI initiation was 17 months in the PPC subgroup compared to 30 months in the GPC subgroup. Finally, only 17% of patients in the PPC subgroup received three or more cycles of FTD-TPI. Statistically significant differences between the GPC and PPC subgroups were only observed in terms of previous lines of therapy (≥3 lines: 78% vs. 17%, *p* = 0.002), time from metastases to FTD-TPI (30 vs. 17 months, *p* = 0.02), and presence of liver metastases (21% vs. 83%, *p* = 0.002).

**Figure 1:**
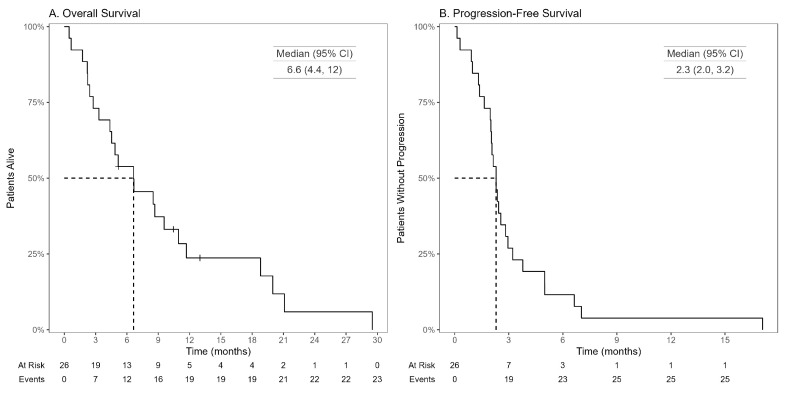
Kaplan–Meier curves for overall survival (**A**) and progression-free survival (**B**) in patients with *BRAF* V600E mutant mCRC treated with FTD-TPI.

**Figure 2:**
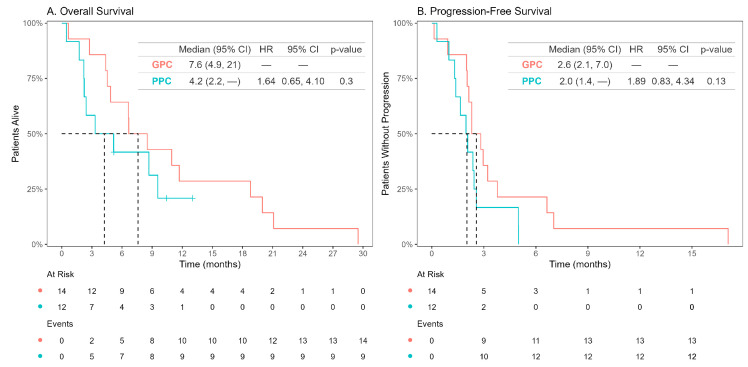
Kaplan–Meier curves for overall survival (**A**) and progression-free survival (**B**) for patients with *BRAF* V600E mutant mCRC treated with FTD-TPI, according to prognostic subgroup (good prognosis characteristics (GPC) and poor prognosis characteristics (PPC)).

In the overall population, median OS (mOS) was 6.6 months (Figure 1A), while median progression-free survival (mPFS) was 2.3 months (Figure 1B). The mOS was higher in the GPC subgroup (Figure 2A), although this difference did not reach statistical significance (GPC mOS: 7.6 months, PPC mOS: 4.2 months; HR 1.64 [95% CI 0.65–4.10, *p* = 0.3]). There was a trend toward longer mPFS among patients in the GPC subgroup (Figure 2B) (GPC mPFS: 2.6 months, PPC mPFS: 2.0 months; HR 1.89 [95% CI 0.83–4.34, *p* = 0.13]).

**Figure 3:**
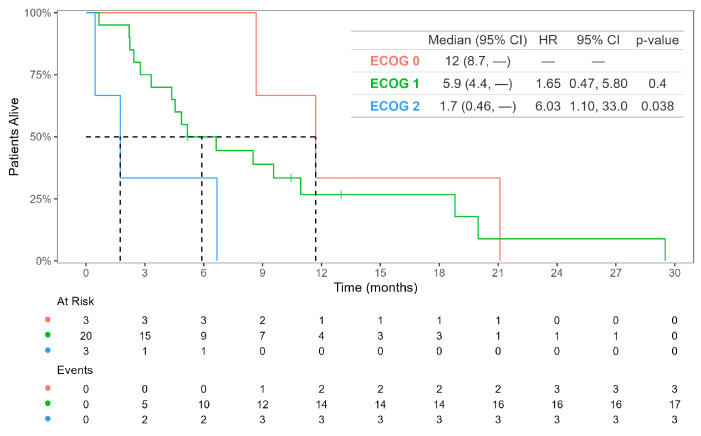
Kaplan–Meier curves for overall survival for patients with *BRAF* V600E mutant mCRC treated with FTD-TPI, according to ECOG performance status.

We evaluated potential prognostic biomarkers, including sex, age at diagnosis; tumor sidedness; previous lines of therapy; MSI status; liver, peritoneal, and lung metastases; ECOG PS; CEA; and CA19.9. Among these factors, only ECOG 2 was associated with survival in a univariate analysis (HR 6.03, 95% CI 1.10–33, *p* = 0.038). The mOS for patients with ECOG 0 was 12.0 months, whereas patients with ECOG 1 and ECOG 2 had mOS values of 5.9 months (HR 1.65, 95% CI 0.47–5.80, *p* = 0.4) and 1.7 months (HR 6.03, 95% CI 1.10–33.0, *p* = 0.038), respectively (Figure 3).

In the overall population, 50% of patients experienced adverse events of any grade, and 19% developed grade 3–4 adverse events. The most common adverse events (any grade) were neutropenia (27%), anemia (23%), asthenia (19%), and nausea (15%). For grade 3–4 toxicities, the most frequent adverse events were neutropenia (8%), anemia (8%), and asthenia (4%). Any-grade toxicity and grade 3–4 toxicities were more frequent among patients in the PPC subgroup (58% vs. 43% and 25% vs. 14%, respectively). Table 2 summarizes toxicities in the overall population and according to prognostic subgroups.

## 4. Discussion

FTD-TPI, with or without bevacizumab, is currently a therapeutic option for patients with mCRC in the refractory setting, regardless of *RAS* and *BRAF* mutational status. Compelling data from two phase 3 randomized trials, the RECOURSE trial (NCT01607957; FTD-TPI vs. placebo) and, more recently, the SUNLIGHT trial (NCT01607957; FTD-TPI-bevacizumab vs. FTD-TPI), demonstrated a survival benefit. However, the efficacy and safety of FTD-TPI either as monotherapy or combined with bevacizumab have not been fully characterized in the *BRAF*-mutant subgroup, which is associated with poor prognosis and aggressive disease. Our real-world cohort is the first to report the clinical activity and toxicity profile in this specific subgroup. In our cohort, while mOS remains modest at 6.6 months, subgroup analysis showed that, among patients without disease-related symptoms or who have ECOG 0, OS can reach 12 months. In our cohort, any-grade toxicity was noted in 50% of patients, whereas grade 3–4 toxicities were observed in 19% of patients. Neutropenia and anemia were the most frequent adverse events (27% and 23%, respectively), but Grade 3–4 neutropenia or anemia occurred in only 8% of patients. In the RECOURSE trial, any-grade toxicity was reported in 98% of patients, and grade 3–4 adverse events were reported in 69% of the population. Neutropenia and anemia (any grade) were reported in 67% and 77% of patients, respectively, with grade 3–4 events occurring in 38% and 18% of patients, respectively. The frequency of adverse events in our cohort was slightly lower than that reported in the literature. However, the small sample size of our cohort and the fact that often, in real-world scenarios, adverse events are not registered in the same way as in clinical trials (particularly if not severe) limit further conclusions. These results suggest that FTD-TPI-based treatment may be clinically active and safe for patients with *BRAF*-mutant CRC.

In recent years, major progress has been made in the development of biomarkers and therapeutic strategies for patients with *BRAF*-mutant mCRC. Tumor load surrogates, such as the BeCool score (which includes ECOG PS, tumor grading, and presence of liver, lung, or nodal metastases) and the plasmatic allele fraction of *BRAF*, have proven to be reliable prognostic biomarkers [14,16]. Various independent cohorts have suggested a predictive value for *RNF43* mutations, although recent research has not fully validated these results [15,26,27]. Furthermore, novel therapeutic strategies, such as the combination of BRAF inhibitors with immune checkpoint inhibitors or rechallenge with a BRAF inhibitor in a well-selected population, have demonstrated promising clinical activity in early clinical trials or case series [28,29,30,31,32]. The combination of BRAF inhibitors with COX-2 inhibitors or anti-VEGF agents has shown encouraging preclinical evidence; however, this has yet to be confirmed in a clinical setting [33,34].

Although new strategies and therapeutic options are being developed, nearly all patients with *BRAF*-mutant CRC will eventually progress to targeted therapy. It is, thus, critical to expand our understanding of available treatments. In such cases, real-world data emerges as a valuable resource, particularly for tumors with low prevalence or an aggressive phenotype, such as *BRAF*-mutant mCRC. These insights can add clinical value and help shape the design of specific clinical trials or novel therapeutic strategies [35]. Real-world data, encompassing information about patient health status and routinely collected during healthcare delivery, serve as a critical resource for generating evidence on care processes and outcomes across the cancer continuum. When subjected to rigorous analytical methodologies, real-world data can address important questions that may be impractical, or infeasible, to explore through clinical trials. However, this key strength of real-world data also presents inherent limitations: real-world databases capture the observed experiences, preferences, and biases of patients, providers, and the healthcare system, often without fully accounting for factors influencing the outcomes. Consequently, real-world evidence—the clinical insights derived from the analysis of real-world data—carries inherent biases and limitations. These must be carefully recognized and methodologically addressed to ensure that robust clinical or policy conclusions can be drawn from the findings [36]. In this context, there are limited real-world data on *BRAF*-mutant colorectal cancer, as it is considered a low-prevalence mutation in colorectal cancer. Regarding this, data from a real-world analysis of 179 patients with *BRAF*-mutant mCRC treated with BRAF inhibitors showed that 53% of patients were unable to receive further treatment after BRAF inhibitor-based therapy due to disease progression and clinical deterioration. Among the 85 patients who did receive additional treatment, only 14 patients were treated with FTD-TPI or regorafenib, and they achieved an mPFS of 2.0 months (range, 1.1–3.4 months) [18]. These results are consistent with those reported in our cohort, which includes a heavily pretreated population with a high tumor burden. Based on previously described prognostic subgroups, we observed that patients with GCP tend to have longer PFS and OS. Additionally, when stratified by ECOG PS, median OS can be improved, with some patients achieving 12-month survival. This study suggests that treatment with FTD-TPI in a selected subgroup of patients with relapsed *BRAF*-mutant CRC and good performance status may significantly prolong survival, providing an additional therapeutic option for this patient population with a poor prognosis.

The retrospective nature of the study and the modest sample size are the main limitations of this research. Furthermore, the SUNLIGHT trial demonstrated the superiority of FTD-TPI combined with bevacizumab over FTD-TPI alone, establishing it as the new standard of care for mCRC, regardless of *RAS/BRAF* mutational status. Given that it is already known that *BRAF*-mutant tumors benefit from the addition of anti-VEGF therapy in combination with chemotherapy, it is likely that FTD-TPI-bevacizumab could further enhance antitumor efficacy in this specific subtype. However, specific data are not currently available, and the findings of the current study with FTD-TPI alone warrant further validation of both single-agent and bevacizumab combination therapy in prospective cohorts.

## 5. Conclusions

In this study, FTD–TPI demonstrated modest OS benefits for patients with *BRAF*-mutant mCRC. Median OS was longer in the GPC group (7.6 months) compared to the PPC group (4.2 months). Additionally, patients with ECOG PS 0 experienced the highest median survival of 12 months, indicating that PS may influence outcomes. Dose modifications were common, and despite 50% of the patients experiencing adverse events, only 19% of these were grade 3–4. While FTD–TPI offers modest clinical benefit, it remains a viable option for select patients, warranting further research in larger cohorts.

## Figures and Tables

**Table 1:** Characteristics of patients with *BRAF* V600E mutant mCRC treated with FTD-TPI overall and according to prognostic characteristics.

		Overall (*n* = 26)	GPC (*n* = 14)	PPC (*n* = 12)	*p* Value
Sex	Male	13 (50)	6 (43)	7 (55)	0.6
	Female	13 (50)	8 (57)	5 (45)	
Age in years, median (range)		61 (51–81)	66 (54–77)	58 (51–88)	
ECOG PS	0	3 (12)	2 (14)	1 (8,3)	0.8
	1	20 (77)	11 (79)	9 (75)	
	≥2	3 (12)	1 (7)	2 (17)	
Sidedness	Right	14 (56)	9 (69)	5 (42)	0.2
	Left	11 (44)	4 (31)	7 (58)	
	NA	1	1	0	
MMR status	MSI	6 (23)	4 (29)	2 (17)	0.7
	MSS	20 (77)	10 (71)	10 (83)	
Previous lines	1	3 (12)	2 (14)	1 (8,3)	0.002
	2	9 (35)	1 (7)	8 (67)	
	3	12 (46)	10 (71)	2 (17)	
	>3	2 (7)	1 (7)	1 (8)	
Prior BRAF inhibitor combination therapy	Yes	18 (70)	10 (71)	8 (67)	0.9
	No	8 (30)	4 (29)	4 (33)	
Prior immunotherapy	Yes	6 (23)	5 (4)	1 (8)	0.17
	No	20 (77)	9 (96)	11 (92)	
Number of metastatic sites	1	4 (15)	4 (29)	0	0.5
	2	13 (50)	10 (71)	3 (25)	
	3	7 (27)	0	7 (58)	
	>3	2 (8)	0	2 (17)	
Liver metastases	Yes	13 (50)	3 (21)	10 (17)	0.02
	No	13 (50)	11 (79)	2 (83)	
CEA, in ng/mL, median (range)		31 (6–2478)	52 (6–2478)	30 (17–55)	0.6
CA19-9, in U/mL, median (range)		123 (23–293)	23 (11–274)	227 (134–291)	0.2
Months from M1 to FTD-TPI, median (range)		29 (18–33)	30 (27–39)	17 (10–32)	0.02
FTD-TPI number of cycles	1	6 (23)	2 (14)	4 (33)	0.8
	2	12 (46)	6 (43)	6 (50)	
	3	2 (8)	1 (7)	1 (8)	
	>3	6 (23)	5 (35)	1 (8)	
FTD-TPI dose modification	Yes	9 (35)	3 (21)	6 (50)	0.2
	No	17 (65)	11 (79)	6 (50)	

All data are presented as the number of patients (percentage) unless otherwise indicated. CEA: carcinoembryonic antigen, ECOG PS: Eastern Cooperative Oncology Group performance status, FTD-TPI: trifluridine/tipiracil, GPC: good prognosis characteristics, M1: metastases, MMR: mismatch repair, MSI: microsatellite instability, MSS: microsatellite stable, NA: not available, PPC: poor prognosis characteristics.

**Table 2:** Summary of the most common adverse events reported in patients with *BRAF* V600E mutant mCRC treated with FTD-TPI, overall and according to prognostic characteristics.

	Overall (*n* = 26)		GPC (*n* = 14)		PPC (*n* = 12)	
	Any Grade	Grade 3–4	Any Grade	Grade 3–4	Any Grade	Grade 3–4
Any adverse event	13 (50%)	5 (19%)	6 (43%)	2 (14%)	7 (58%)	3 (25%)
Neutropenia	7 (27%)	2 (8%)	5 (36%)	1 (7%)	2 (17%)	1 (8%)
Anemia	6 (23%)	2 (8%)	3 (21%)	1 (7%)	3 (25%)	1 (8%)
Asthenia	5 (19%)	1 (4%)	2 (14%)	0 (0%)	3 (25%)	1 (8%)
Nausea	4 (15%)	0 (0%)	1 (7%)	0 (0%)	3 (25%)	0 (0%)
Abdominal pain	2 (8%)	0 (0%)	1 (7%)	0 (0%)	1 (8%)	0 (0%)
Stomatitis	2 (7.7%)	0 (0%)	0 (0%)	0 (0%)	2 (16.7%)	0 (0%)
Thrombocytopenia	2 (7.7%)	0 (0%)	1 (7.1%)	0 (0%)	1 (8.3%)	0 (0%)
Vomiting	2 (7.7%)	0 (0%)	1 (7.1%)	0 (0%)	1 (8.3%)	0 (0%)
ALT elevation	1 (3.8%)	0 (0%)	1 (7.1%)	0 (0%)	0 (0%)	0 (0%)
AST elevation	1 (3.8%)	0 (0%)	1 (7.1%)	0 (0%)	0 (0%)	0 (0%)
Bilirubin increased	1 (3.8%)	0 (0%)	0 (0%)	0 (0%)	1 (8.3%)	0 (0%)
Diarrhea	1 (3.8%)	0 (0%)	0 (0%)	0 (0%)	1 (8.3%)	0 (0%)

ALT: alanine transaminase, AST: aspartate transaminase, GPC: good prognosis characteristics, PPC: poor prognosis characteristics.

## Data Availability

Clinical data from the cohort will be made available upon reasonable request and can be obtained by contacting us via email.

## Supplementary Materials

The following supporting information can be downloaded at: https://www.mdpi.com/article/10.3390/cancers16244140/s1, ESMO-GROW Checklist for Authors and Reviewers [37].
